# Compatibility of different *Metarhizium* spp. propagules with synthetic acaricides for controlling *Rhipicephalus microplus*

**DOI:** 10.1590/S1984-29612022018

**Published:** 2022-04-01

**Authors:** Adriani da Silva Carneiro, Emily Mesquita, Laura Nóbrega Meirelles, Vânia Rita Elias Pinheiro Bittencourt, Patrícia Silva Golo

**Affiliations:** 1 Programa de Pós-graduação em Ciências Veterinárias, Instituto de Veterinária, Universidade Federal Rural do Rio de Janeiro – UFRRJ, Seropédica, RJ, Brasil; 2 Departamento de Parasitologia Animal, Instituto de Veterinária, Universidade Federal Rural do Rio de Janeiro – UFRRJ, Seropédica, RJ, Brasil

**Keywords:** Entomopathogenic fungi, ticks, conidia, blastospores, microsclerotia, Fungos entomopatogênicos, carrapatos, conídios, blastosporos, microescleródios

## Abstract

The inappropriate use of synthetic acaricides has selected resistant *Rhipicephalus microplus* populations. The present study evaluated the compatibility of different *Metarhizium* spp. propagules (conidia, blastospores, and microsclerotia) by incubating them with synthetic acaricides (amitraz, deltamethrin, and a combination of cypermethrin, chlorpyrifos, and citronellal) for 1 h, 5 h, 10 h, and 24 h. Conidia and microsclerotia of the tested isolates were usually more tolerant to synthetic acaricides than blastospores. Our study also analyzed the *in vitro* effect of deltamethrin associated with fungal propagules for controlling a population of *R. microplus* females that were not susceptible to this synthetic acaricide. The use of entomopathogenic fungi in association with deltamethrin in this tick population caused a greater tick control than did the use of the fungus or the synthetic acaricide separately.

## Resumo

O uso inadequado de acaricidas sintéticos selecionou populações resistentes de *Rhipicephalus microplus*. O presente estudo avaliou a compatibilidade de diferentes propágulos de *Metarhizium* spp. (conídios, blastosporos e microscleródios), incubando-os com acaricidas sintéticos (amitraz, deltametrina e uma combinação de cipermetrina, clorpirifós e citronelal) por 1 h, 5 h, 10 h e 24 h. Conídios e microescleródios dos isolados testados foram geralmente mais tolerantes a acaricidas sintéticos do que os blastosporos. Este estudo também analisou o efeito *in vitro* da deltametrina associada a propágulos fúngicos no controle de uma população de fêmeas de *R. microplus* não suscetíveis a este acaricida sintético. O uso de fungos entomopatogênicos em associação com deltametrina, nesta população de carrapatos, causou maior controle do carrapato do que o uso do fungo ou acaricida sintético isoladamente.

## Introduction

*Rhipicephalus microplus* is a blood-sucking ectoparasite. Infestations with this tick are common on cattle in tropical areas. *R. microplus* control strategies involve the use of synthetic acaricides, and the main classes used are organochlorines, synthetic pyrethroids, organophosphates, amidines, phenylpyrazoles, growth regulators, and avermectins (Rodriguez-Vivas et al., 2018). Indiscriminate use of these acaricides and inappropriate application methods have been selecting resistant tick populations, thus driving livestock producers to increase the frequency of application, which negatively impacts the safety of meat and milk (Kumar et al., 2016). Using fungi and chemical acaricides concomitantly have become a more sustainable and interesting approach, with the aim of reducing the selective pressure and other negative impacts caused by these chemicals (Webster et al., 2015).

Entomopathogenic fungi can naturally produce different types of propagules during their life cycle, including conidia and hyphal bodies (Alkhaibari et al., 2016). Production of a third propagule type, known as microsclerotia, can be artificially induced. Conidia are structures slightly larger than nine µm in length that is involved in the dispersion of entomopathogenic fungi. They are the type of propagule most used in bioproducts for pest control (Mascarin et al., 2019). Conidia have high resistance and high capacity for adherence and penetration into the arthropod host's cuticle. Blastospores are hydrophilic, pleomorphic vegetative structures that are considered similar to the hyphal bodies found in arthropods’ hemocele when produced in a 48 to 72-hour liquid culture medium. They usually germinate faster than conidia and can be viable for several months, depending on how they are stored (Bernardo et al., 2018; Mascarin et al., 2019). Microsclerotia are commonly produced by phytopathogenic fungi and improve the persistence of these fungi in the soil (Coley-Smith & Cooke, 1971). Although microsclerotia cannot be naturally produced by entomopathogenic fungi, *Metarhizium* can be induced to make them and is considered to be a promising propagule for the development of mycoinsecticides and mycoacaricides (Marciano et al., 2021).

Most mycoinsecticides currently produced in Brazil are based on aerial conidia that are harvested after solid substrate fermentation (Mascarin et al., 2019). On the other hand, blastospores and microsclerotia can be mass-produced by liquid fermentation, which represents an advantage for the industry in terms of reduced time, cost, and labor (Mascarin et al., 2019). Recent studies on entomopathogenic fungal propagules other than conidia have shown their potential for use in tick control (Bernardo et al., 2018; Marciano et al., 2021). Studies addressing fungal compatibility with synthetic acaricides are greatly needed but, as far as we know, there is no literature on the compatibility of blastospores and microsclerotia of *Metarhizium* spp. and synthetic acaricides, and concomitant use of these methods for controlling cattle ticks. Accordingly, the present study evaluated the compatibility of different propagules of *Metarhizium* spp. (i.e., conidia, blastospores, and microsclerotia) with synthetic acaricides and their *in vitro* effect against engorged females of *R*. *microplus*.

## Material and Methods

### Tick and fungal isolates

The ticks used in the present study were artificially obtained (CEUA / IV / UFRRJ protocol no. 9714220419). The fungal isolates used here were *Metarhizium robertsii* isolate ARSEF 2575, which was obtained from the Agricultural Research Service Collection of Entomopathogenic Fungal Cultures (ARSEF; USDA Plant, Soil, and Nutrition Laboratory, United States) and *Metarhizium pingshaense* isolate LCM S09 (Mesquita et al., 2020). Because the present study accessed Brazilian genetic heritage, the research was registered in the National System for the Management of Genetic Heritage and Associated Traditional Knowledge (SISGEN) under the code AA47CB6.

### Compatibility of *Metarhizium* spp. conidia with commercial acaricides

Conidia of ARSEF 2575 and LCM S09 were produced on oatmeal agar (OA), from 60 g of oat flour added to 12.5 g of agar in 1 liter of distilled water, at 25 °C ± 1 °C and relative humidity (RH) ≥ 80% for 14 days. The compatibility of these conidia with acaricides was evaluated using three commercial products: amitraz (12.5%, Triatox^®^, MSD, São Paulo, Brazil), deltamethrin (2.5%, Butox^®^, MSD, São Paulo, Brazil) and a mixture of pyrethroid, organophosphate and monoterpenoid alcohol (15% cypermethrin, 25% chlorpyrifos and 1% citronellal; Colosso^®^, Ourofino, São Paulo, Brazil). Each commercial acaricide was added to *Metarhizium* conidial Tween 80^®^ suspensions (final concentration of 10^3^ conidia ml^-1^) and was kept in contact for four different periods (1 h, 5 h, 10 h, and 24 h). The final concentrations of the acaricides followed what is recommended by their manufacturers for spraying on cattle (i.e., 0.025% for amitraz; 0.0025% for deltamethrin; and 0.018% for cypermethrin + 0.031% for chlorpyrifos + 0.0012% for citronellal). Tween 80^®^ sterile distilled water (0.01%) was added to the fungal suspensions in the control groups. The suspensions were then plated onto OA and left at 25 °C ± 1 °C and RH ≥ 80% for 96 h (Webster et al., 2015). Colony-forming units (CFUs) were quantified and the relative culturability was calculated as prescribed by Braga et al. (2001). Experiments were considered valid when control plates contained at least 95% germinated conidia. The experiments were performed three times with different batches of conidia.

### Compatibility of *Metarhizium* spp. blastospores with commercial acaricides

For blastospores production, *Metarhizium* spp. conidia were grown on OA (25 °C ± 1 °C; RH > 80%) for 14 days. Three milliliters of conidial suspensions (10^8^ conidia ml^-1^) were inoculated into 50 ml of oatmeal broth (60 g of oat flour and 42.1 gl^-1^ of glucose) at 200 rpm for 72 h at 25 °C ± 1 °C. The blastospores were produced as described by Corval et al. (2021). The suspensions were adjusted to 2.0 × 10^3^ blastospores ml^-1^. Each commercial acaricide was added to a *Metarhizium* blastospores suspension (final concentration of 10^3^ blastospores ml^-1^) and was left in contact for four different time periods, following the methodology described for conidia. CFUs were quantified and the relative culturability was calculated as described by Braga et al. (2001). Experiments were considered valid when control plates contained at least 95% germinated blastospores. The experiments were performed three times with different batches of blastospores.

### Compatibility of *Metarhizium* spp. microsclerotia with commercial acaricides

Microsclerotia from *Metarhizium* spp. were prepared as described by Mascarin et al. (2014). Each commercial acaricide was added to 3 ml of *Metarhizium* microsclerotia suspensions (final concentration of ~300 microsclerotia ml^-1^), following the methodology described for conidia. After the incubation with the synthetic acaricides, an aliquot of 30 microsclerotia was transferred to an agar-agar medium. Germination was assessed at 72 h. Microsclerotia granules were considered to have germinated upon observation of hyphal development around the granule. Experiments were considered valid when control plates contained at least 95% germinated microsclerotia. The relative culturability was calculated as previously described by Braga et al. (2001). The experiments were performed three times with different batches of microsclerotia.

### Biological assays with engorged females of *Rhipicephalus microplus*

Completely engorged females were distributed in 14 experimental groups. These ticks were treated with an association of deltamethrin (Butox^®^) and *M. robertsii* or *M. pingshaense* propagules (i.e., conidia, blastospores, or microsclerotia). The fungal propagules had not previously been incubated with the synthetic acaricide. The positive control group was treated either with the fungal propagules or the chemical acaricide alone. In the negative control group, the females were treated with Tween 80^®^ aqueous suspension at 0.01% (vol/vol).

Each group comprised 10 *R. microplus* females, which were individually weighed and homogenously distributed into the groups. The ticks that received treatment with conidia or blastospores were immersed in 1 ml of fungal suspensions at 10^8^ propagules ml^-1^ for three minutes. For microsclerotia, females were immersed in a suspension of 100 microsclerotia per tick for three minutes. Fungal suspensions were added to the synthetic acaricide solution and the same fungal concentrations previously described were used for the association treatments. The final concentrations of the acaricides followed what is recommended by their manufacturers for spraying on cattle.

The methodology that was used to treat the engorged females followed the descriptions of Perinotto et al. (2017). The biological parameters evaluated were egg mass weight (EMW), larval hatching percentage (LH), egg production index (EPI), and nutritional index (NI). The percentage of tick control was obtained by calculating the estimated reproduction, as described by Drummond et al. (1971). Bioassays were performed twice.

### Statistical analysis

Data from the compatibility tests (associations of fungi and synthetic acaricides) were transformed (√x + 0.5) and subjected to the Shapiro-Wilk normality test and Bartlett homogeneity of variance test. After these assumptions had been assessed, two-way ANOVA and the Tukey test (P < 0.05) were applied to compare these treatments. The biological parameters (i.e., EMW, LH, EPI, and NI) exhibited non-normal distribution. Data with non-normal distribution were evaluated using the Kruskal-Wallis test followed by Dunn's test (P < 0.0001). All data were analyzed using the GraphPad Prism software, v.9.1.2.

## Results

### Compatibility of *Metarhizium* spp. propagules with chemical acaricides

**Figure 1:**
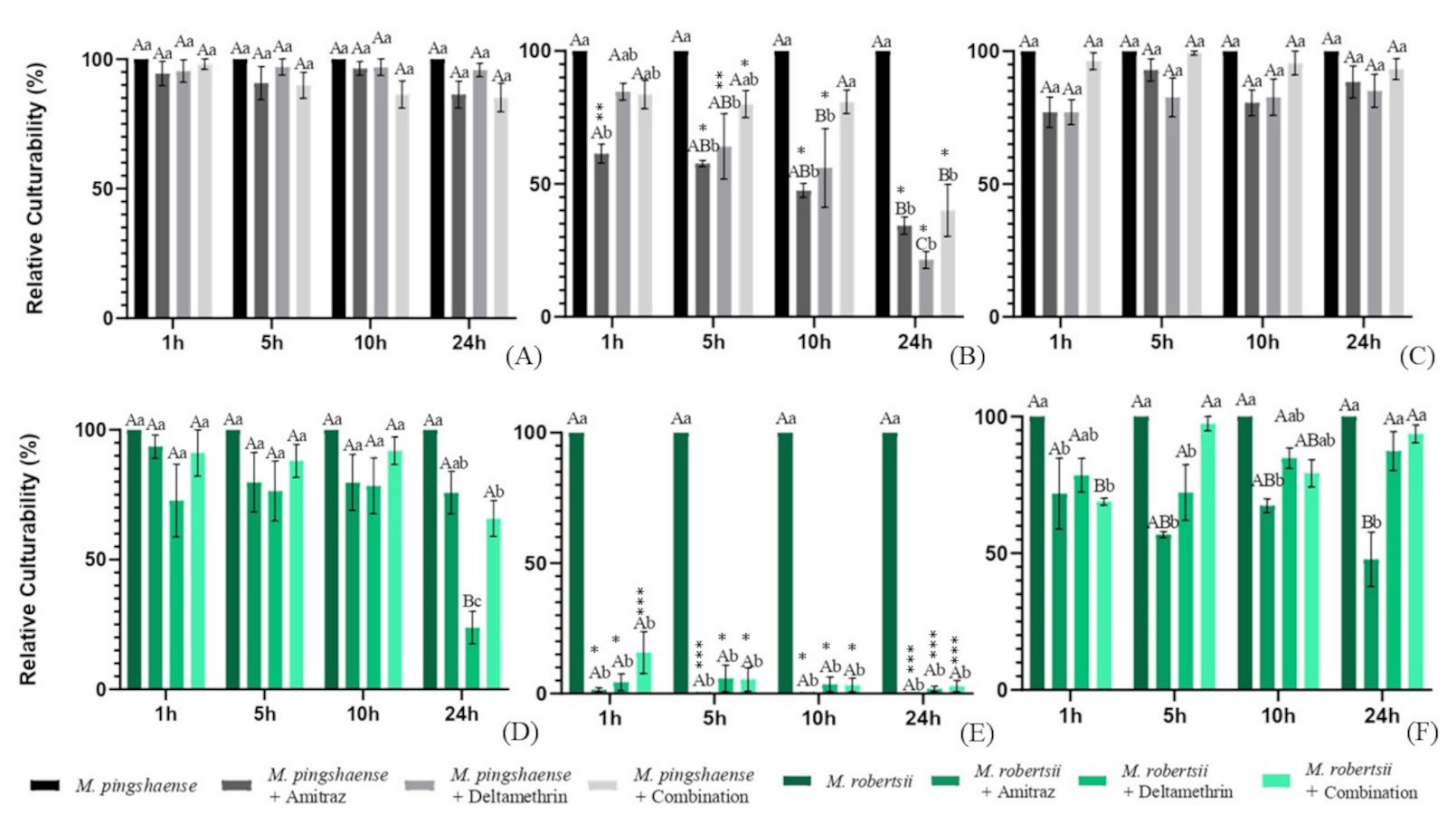
Mean and standard error of relative culturability (%) of (**A**) *Metarhizium pingshaense* LCM S09 conidia, (**B**) blastospores, and (**C**) microsclerotia, or (**D**) *Metarhizium robertsii* ARSEF 2575 conidia, (**E**) blastospores, and (**F**) microsclerotia associated or not with amitraz (Triatox^®️^), deltamethrin (Butox^®️^) or combination of acaricides [Chlorpyrifos, cypermethrin, and citronellal (Colosso^®️^)] for 1 h, 5 h, 10 h, and 24 h. Bars with the same uppercase letter in the same treatment, propagule, and fungal isolate or the same lowercase letter in the same incubation period, propagule, and fungal isolate did not differ statistically (P > 0.05). Asterisks indicate a statistical difference in the relative culturability of different propagules from the same fungal isolate that were exposed to the same treatment considering the same incubation period (P < 0.05). (*) Indicates significant difference in the relative culturability of blastospores in comparison to conidia and microsclerotia. (**) Indicates significant difference in the relative culturability of blastospores and microsclerotia in comparison to conidia. (***) Indicates that the relative culturability of all propagules was different from each other.

The relative culturabilities of *Metarhizium* spp. conidia, blastospores and microsclerotia exposed to the different acaricides for the different lengths of time are shown in Figure 1. The relative culturability of conidia ranged from 98% (*M. pingshaense* conidia + combination; 1 h) to 23.8% (*M. robertsii* conidia + deltamethrin; 24 h). With blastospores, it ranged from 83.6% (*M. pingshaense* blastospores + combination; 1 h) to 0.7% (*M. robertsii* blastospores + amitraz; 24 h). The relative culturability of microsclerotia ranged from 99.3% (*M. pingshaense* microsclerotia + combination; 5 h) to 47.8% (*M. robertsii* microsclerotia + amitraz; 24 h). 24 h of incubation with acaricides negatively impacted the culturability of *M. robertsii* conidia and microsclerotia, but not that of *M. pingshaense* (Figure 1A and 1C). Significant differences were observed in the relative culturabilities of different propagules that were exposed to the same treatment considering incubation for 1 h (propagules: F_2,48_ = 94.7 and P < 0.0001; treatments: F_7,48_ = 39 and P < 0.0001); 5 h (propagules: F_2,48_ = 113.2 and P < 0.0001; treatments: F_7,48_ = 39.8 and P < 0.0001); 10 h (propagules: F_2,48_ = 146.5 and P < 000.1; treatments: F_7,48_ = 40.3 and P < 0.0001); and 24h (propagules: F_2,48_ = 228.3 and P < 0.0001; treatments: F_7,48_ = 67.85 and P < 0.001). Asterisks were included in Figure 1 to address the differences in the relative culturabilities of different propagules from the same fungal isolate that were exposed to the same treatment and time of incubation.

### Biological assays with engorged females of *Rhipicephalus microplus*

Table 1 shows the results from female tick immersion tests using different fungal propagules and deltamethrin. EMW ranged from 15.4 mg to 170.2 mg and LH from 71.1% to 95.7% in the treated groups, while in the untreated group (CTR), values of 158.7 mg (EMW) and 97.3% (LH) were observed. The EPI and NI yielded ranged from 5.8% to 61.0% and 11.1% to 79.6% respectively. The highest tick control percentage observed was 81.5%, in the treatment with *M. pingshaense* conidia and deltamethrin.

**Table 1:** Mean values ± standard error of egg mass weight (EMW), larval hatch percent (LH), egg production index (EPI), nutritional index (NI), and control percent of *Rhipicephalus microplus* females treated with different *Metarhizium* spp. propagules associated or not with deltamethrin.

**Groups**	**EMW (mg)**	**LH (%)**	**EPI (%)**	**NI (%)**	**Tick Control (%)**
**Untreated (CTR)**	158.7 ± 0 a	97.3 ± 2 a	57.5 ± 4 a	77.6 ± 3.4 ab	
	(Md = 0.2)	(Md = 100)	(Md = 62.6)	(Md = 81.3)	
	-10	-10	-10	-10	
			
**Deltamethrin**	161.7 ± 0 a	86.4 ± 9.8 ab	58.4 ± 2.1 a	79.3 ± 3.4 ab	7.23
	(Md = 0.1)	(Md = 97)	(Md = 59.5)	(Md = 81.3)	
	-10	-10	-10	-10	
***M. pingshaense* (conidia)**	110.6 ± 0 bc	77.0 ± 9 bc	39.8 ± 3.4 bc	64.2 ± 3.7 bc	41.88
	(Md = 0.1)	(Md = 85)	(Md = 41.5)	(Md = 65.6)	
	-10	-10	-10	-10	
***M. pingshaense* (blastospores)**	149.1 ± 0 abc	95.7 ± 1.1 ab	53.8 ± 2.2 ac	68.7 ± 2.7 abc	8.57
	(Md = 0.1)	Md = 96.5)	(Md = 55.9)	(Md = 68.2)	
	-10	-10	-10	-10	
***M. pingshaense* (microsclerotia)**	165.9 ± 0 a	83.0 ± 2.5 bc	60.2 ± 0.9 a	76.5 ± 1.6 ab	11.1
	(Md = 0.2)	(Md = 80)	(Md = 60.6)	(Md = 77.4)	
	-10	-10	-10	-10	
***M. robertsii* (conidia)**	138.4 ± 0 abc	93.2 ± 1.5 abc	49.6 ± 5.7 ac	63.57 ± 7.2 ac	8.23
	(Md = 0.1)	(Md = 95.0)	(Md = 55.3)	(Md = 69.6)	
	-10	-9	-10	-10	
***M. robertsii* (blastospores)**	170.2 ± 0 a	89.50 ± 4.3 abc	61.0 ± 1 a	79.6 ± 1.6 a	2.53
	(Md = 0.2)	(Md = 98)	(Md = 60.2)	(Md = 79.8)	
	-10	-10	-10	-10	
***M. robertsii* (microsclerotia)**	165.2 ± 0 a	85 ± 4.4 abc	59.5 ± 1.5 a	74.4 ± 1.4 ab	10.27
	(Md = 0.2)	(Md = 89)	(Md = 60)	(Md = 74)	
	-10	-10	-10	-10	
***M. pingshaense* (conidia) + Deltamethrin**	15.4 ± 0 c	90.6 ± 3 abc	5.8 ± 2.6 bc	11.1 ± 4.4 c	81.15
	(Md = 0)	(Md = 90)	(Md = 2.1)	(Md = 4.6)	
	-10	-5	-10	-10	
***M. pingshaense* (blastospore) + Deltamethrin**	141.2 ± 0 abc	93.2 ± 1.8 abc	50.4 ± 2.6 ac	66.3 ± 2 abc	16.04
	(Md = 0.1)	(Md = 95)	(Md = 51.5)	(Md = 68.40)	
	-10	-10	-10	-10	
***M. pingshaense* (microsclerotia) + Deltamethrin**	126.0 ± 0 abc	71.1 ± 5.6 c	45.6 ± 6 ac	59.7 ± 7.5 abc	34.36
	(Md = 0.1)	(Md = 80)	(Md = 53.4)	(Md = 67.3)	
	-10	-9	-10	-10	
***M. robertsii* (conidia) + Deltamethrin**	148.0 ± 0 abc	94.7 ± 1.1 abc	53.48 ± 1.1 ac	64.8 ± 1.3 c	9.84
	(Md = 0.1)	(Md = 95)	(Md = 54.2)	(Md = 64.5)	
	-10	-10	-10	-10	
***M. robertsii* (blastopores) + Deltamethrin**	141.7 ± 0 ab	86.8 ± 4 abc	50.7 ± 5.7 ac	62.73 ± 7.2 abc	12.84
	(Md = 0.2)	(Md = 85)	(Md = 55)	(Md = 67.7)	
	-10	-9	-10	-10	
***M. robertsii* (microsclerotia) + Deltamethrin**	153.1 ± 0 ab	88.2 ± 4 abc	55 ± 0.7 ac	62.7 ± 1.6 abc	13.71
	(Md = 0.1)	(Md = 95)	(Md = 54.9)	(Md = 70.2)	
	-10	-10	-10	-10	

Mean values followed by the same letter in the same column do not differ statistically (P > 0.05). Md = median. (n) Sample size.

## Discussion

Resistance of *R. microplus* to acaricides is an issue that has been observed in several countries around the world and it occurs with different classes of acaricides. The intensity and frequency of use of these products and their misuse are factors that greatly contribute to occurrences of resistance. The use of entomopathogenic fungi is considered to be a promising alternative for tick control (Rodriguez-Vivas et al., 2018). Nonetheless, biological products can be negatively impacted by unfavorable environmental and storage conditions, abiotic factors, host ecology, and the presence of other fungi in the soil (saprophytic fungi) (Mascarin et al., 2019). Some studies have reported that associations of entomopathogenic fungi with acaricides have potential applicability for controlling *R. microplus*, thereby enabling the use of chemical bases that had previously been less effective against some tick populations and leading to reduction of the selective pressure on other bases. Through this, occurrences of resistance to chemical acaricides can be weakened (Bahiense & Bittencourt, 2004; Webster et al., 2015).

Entomopathogenic fungi actively penetrate the host’s cuticle and proliferate inside the organism causing the arthropod’s death. Here, the culturability of *Metarhizium* conidia and microsclerotia was less impacted than that of blastospores, when used in combination with the acaricides (Figure 1). Conidia are produced by phialides (in a process called conidiogenesis), while microsclerotia are clusters of melanized hyphae that are highly resistant to oxidative stress and capable of producing microbial compounds, and blastospores are vegetative structures produced by hyphal budding that gives them a weaker membrane (Hallsworth & Magan, 1996; Song et al., 2016; Kim et al., 2013; Jackson & Payne, 2016; Bernardo et al., 2020; Corval et al., 2021). It is suggested that differences in the physical structures of propagules can impact their susceptibility to synthetic acaricides and this assumption is supported by our results.

The use of entomopathogenic fungi in association with synthetic acaricides, depending on the chemical base and solvent, can negatively impact fungal germination and mycelial growth (Mohamed et al., 1987; Batista et al., 2001). Moreover, different *Metarhizium* isolates have been found to exhibit different germination and growth patterns when synthetic acaricides were incorporated into the artificial media (Schumacher & Poehling, 2012). In the present study, there was no inconvenience when incorporating the fungal propagules with the synthetic acaricides, i.e., the solvents for the acaricides and their active ingredients could be homogeneously mixed with the fungal propagule suspensions. Here, *M. pingshaense* (LCM S09) blastospores were overall more tolerant than those of *M. robertsii* (ARSEF 2575) (Figure 1). Also, *M. pingshaense* LCM S09 conidia and microsclerotia had higher relative culturability 24 h after incubation with some synthetic acaricides than what was observed for *M. robertsii* ARSEF 2575 (Figure 1).

Our results revealed that the use of entomopathogenic fungi in association with synthetic acaricides (considering both their active ingredients and solvents) can represent a stressful condition for these fungi, particularly for some propagules and isolates when incubated for long periods. The distinct levels of culturability observed in the present study suggest that the isolates tested have different capacities to overcome stressful situations. This is important with regard to framing the strategies for integrated pest management. As reported in the literature, blastospores can be effective propagules against ticks (Bernardo et al., 2018), but if synthetic acaricides are used concomitantly to these propagules, it is not recommended to incubate both in combination. It is important to mention that here when propagules were plated onto the culture medium, after the incubation periods, they still could be in contact with the acaricides’ residues. Despite this, some propagules were able to germinate and grow (Figure 1) simulating what happens on the arthropod’s surface.

*M. pingshaense* LCM S09 yielded higher tick control percentages than did *M. robertsii* ARSEF 2575. Moreover, when the tick control percentages of *M. pingshaense* propagules were compared, conidia had better results. The tick population used in the present study was not susceptible to deltamethrin (Table 1), but to amitraz (Triatox^®^) or to the combination of acaricides (Colosso^®^) (unpublished results). That is why these synthetic acaricides were not used in the bioassay. Application of deltamethrin plus *M. pingshaense* LCM S09 conidia to ticks that were susceptible to this synthetic acaricide caused greater tick control than the fungus or the synthetic acaricide alone (Table 1). This result was also observed by Bahiense & Bittencourt (2004). Nevertheless, here, not all propagules of the same fungal isolate exhibited a synergic effect, particularly if the isolate was not virulent to the tick. In the present study, EPI and NI were statistically similar for most treatments, even though some groups exhibited considerable numerical variation (viz., *M. pingshaense* conidia + deltamethrin and *M. robertsii* microsclerotia + deltamethrin) (Table 1).

Microsclerotia has been reported to be amenable to dry granular formulations that can be used in the field for tick control (Marciano et al., 2021). These granules can be scattered on the soil and upon rehydration, microsclerotia can form infective conidia *in situ* (Mascarin et al., 2019). As far as we know, the present study is the first report of the direct application of microsclerotia propagules on *R. microplus* ticks. Our results show that the immersion of tick females into a suspension of *Metarhizium* microsclerotia can negatively impact the reproductive parameters of ticks (Table 1). Nevertheless, our study did not investigate if microsclerotia can directly infect the tick or if these propagules must produce conidia on the host cuticle, and these conidia are the real ones responsible for the infection.

In the present study, tick females were immersed in suspensions of fungal propagules and deltamethrin. These suspensions had not been incubated previously (i.e., ticks were immediately treated with the suspension of fungus plus deltamethrin right after the suspension assembly). Accordingly, the effect of the incubation of fungal propagules plus synthetic acaricides on tick control was not evaluated. Despite this, based on the results from the compatibility tests, it can be suggested that even if *M. pingshaense* LCM S09 conidia or microsclerotia and deltamethrin were incubated for up to 24 h, this incubation would not negatively impact the effectiveness of these propagules against ticks. The same should not be suggested for blastospores, since their culturability was affected by the incubation with the synthetic acaricides (Figure 1).

## Conclusion

*Metarhizium* spp. conidia and microsclerotia were more compatible with synthetic acaricides than blastospores. The use of virulent entomopathogenic fungus plus deltamethrin gave rise to greater tick control than using the fungus or the acaricide separately in a population of ticks that were not susceptible to deltamethrin.
